# New Insights on the Composition and the Structure of the Acellular Extrinsic Fiber Cementum by Raman Analysis

**DOI:** 10.1371/journal.pone.0167316

**Published:** 2016-12-09

**Authors:** Thomas Colard, Guillaume Falgayrac, Benoit Bertrand, Stephan Naji, Olivier Devos, Clara Balsack, Yann Delannoy, Guillaume Penel

**Affiliations:** 1 Univ. Lille, CHU Lille, EA 7367—UTML—Unité de Taphonomie Médico-Légale, Lille, France; 2 Univ. Lille, EA 4490—PMOI–Physiopathologie des Maladies Osseuses Inflammatoires, Lille, France; 3 CIRHUS-NYU, New York City, NY, United States of America; 4 Univ. Lille, CNRS, UMR 8516—LASIR—Laboratoire de Spectrochimie et Raman, Lille, France; Tufts University, UNITED STATES

## Abstract

Acellular extrinsic fiber cementum is a mineralized tissue that covers the cervical half of the tooth root surface. It contains mainly extrinsic or Sharpey’s fibers that run perpendicular to the root surface to anchor the tooth via the periodontal ligament. Acellular cementum is continuously and slowly produced throughout life and exhibits an alternating bright and dark pattern under light microscopy. However, although a better understanding of the structural background of acellular cementum is relevant to many fields, such as cementochronology, periodontology and tissue engineering, acellular cementum remains rarely studied and poorly understood. In this work, we studied the acellular cementum at the incremental line scale of five human mandibular canines using polarized Raman spectroscopy. We provided Raman imaging analysis and polarized acquisitions as a function of the angular orientation of the sample. The results showed that mineral crystals were always parallel to collagen fibrils, and at a larger scale, we proposed an organizational model in which we found radial collagen fibers, “orthogonal” to the cementum surface, and “non-orthogonal” fibers, which consist of branching and bending radial fibers. Concerning the alternating pattern, we observed that the dark lines corresponded to smaller, more mineralized and probably more organized bands, which is consistent with the zoological assumption that incremental lines are produced during a winter rest period of acellular cementum growth.

## I. Introduction

Cementum is an avascular and not innerved mineralized tissue that covers the tooth root surface and is continuously and slowly produced throughout life. Contrary to bone tissue, it is not subject to remodeling processes [1]. Cementum is generally classified into five different categories, which are mainly based on the presence/absence of cementocytes and the nature (extrinsic and/or intrinsic) of the collagen fibers [2–4]. The three main types are the Cellular Intrinsic Fibers Cementum (CIFC), the Acellular Extrinsic Fibers Cementum (AEFC) and the Mixed Stratified Cementum, which exhibits lines of cellular and acellular cementum.

The cellular cementum is relatively thick, contains both extrinsic and intrinsic collagen fibers and covers the apical half of the root. The acellular cementum is generally considered to contain only extrinsic or Sharpey’s fibers and covers the cervical half of the root. Its main role is to anchor the tooth via the periodontal ligament (PDL). However, Ho et al. [4] demonstrated that distribution pattern of cellular and acellular cementum may be different (i.e. cellular cementum from the cervical to apical portion) but mainly in case of hypercementosis.

In human cementum, collagens fibers run in two distinct orientations: radial fibers (acellular extrinsic or Sharpey’s fibers) and circumferential (cellular intrinsic fibers). Extrinsic fibers predominantly run perpendicular to the root surface, continuous to the cement-dentin junction, although some of them may intermingle, branch or bend and thus appear parallel to the root surface [5–7]. In terms of growth, Aboulfadl et al. [8] demonstrated by PS-SHGM analysis that cementum is growing in three directions: radial fibers grow toward the surface (D-groups of collagen fibrils point to the surface), although circumferential fibers show 180° orientational disorder and grow in two opposite directions. AEFC growth can be described as the progressive mineralization of PDL Sharpey’s fibers under the control of pyrophosphate [9].

Biochemical analyses have shown that, similar to other calcified tissues, approximately 60–65% of AEFC is inorganic and consists of nano-sized mineral particles, such as hydroxyapatite crystals (Ca_10_(PO_4_)_6_(OH)_2_), with small amounts of amorphous calcium phosphates. The remaining organic matrix is primarily composed of type 1 collagen, which provides scaffolding for mineral crystals [10].

However, cementum remains a relatively poor studied and understood tissue, and most previous studies have been concerned with the cellular cementum [i.e 11–13]. Further, the AEFC structural background deserves to be further understood because it is a key factor in very different areas, such as tissue engineering in dental implant surgery, cementum regeneration or estimating the age at death for zoologists or anthropologists.

Under light microscopy, the AEFC shows an alternating bright and dark pattern. Some studies have investigated whether the cementum layering pattern is produced by collagen fibers that change their orientations [11–17] or crystallite direction [18], have different mineralization patterns [16, 19–22], contain collagen packing as in bone [23,24] or exhibit variations in their organic or inorganic matrices [20,25]. The absence of remodeling and the assumption of continuous and slow growth materialized by the specific alternating pattern have attracted interest from zoologists [26], human biologists [27,28], bioarchaeologists [29–31] and forensic anthropologists [32]. Indeed, counting the cementum incremental lines is widely considered a good method of estimating the calendar age of an individual. Even given the physiological variations in AEFC growth related to individual life histories, the effects of exposure to particular biomechanical constraints and pathogens on the cementum remain poorly understood and must impact the accuracy and precision of age-at-death estimation studies. Most previous studies of animals and humans assume a superior performance by cementochronology [28,31,33]. Behind this consensus regarding the method’s effectiveness and the well-known biochemical composition of cementum, an obstacle to the adoption of this technique is hidden, as underlined by Renz and Radlanski in 1997 [34], who noted the unclear physiological and structural biological background of cementum.

A better understanding of the structural background of AEFC is also relevant in the field of tissue engineering, particularly for creating a biomimetic periodontal ligament around titanium implant surfaces [7].

This paper aims to investigate AEFC at the alternating cementum incremental lines scale on human teeth using Raman Spectrometry (RS). Raman spectroscopy has the definitive advantage of providing crucial information about the composition and the structure of AEFC. Numerous studies have demonstrated the efficiency of RS in observing simultaneous orientation oscillations in collagen fibrils and mineral crystals at the lamellar scale and modifications of the composition or structure of biological mineralized tissues [35–38].

## II. Results

### 2.1 Chemical composition of acellular extrinsic fiber cementum

**Fig 1:**
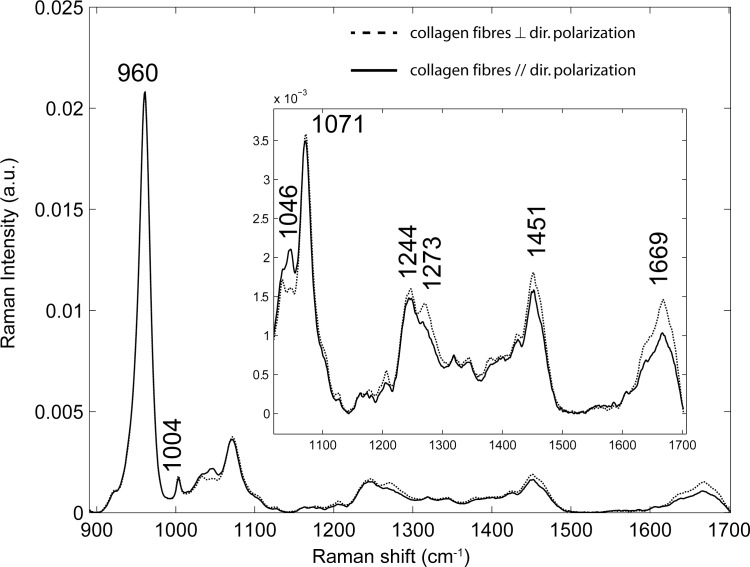
Polarized Raman spectra of AEFC Raman spectra of AEFC as a function of the angle between the long axis of collagen mineralized fibres (collagen fibres) and the direction of polarization (dir. polarization). The Raman spectrum in continuous line was observed when the long axis of mineralized collagen fibres and the direction of polarization were parallels. The Raman spectrum in dash line was observed when the long axis of mineralized collagen fibres and the direction of polarization were perpendiculars.

The Raman spectra in Fig 1 are representative of AEFC in cross- and longitudinal sections. In each section, Raman spectra were acquired from the bright and dark lines, and similar spectra between those incremental lines were revealed in all samples. The assignment of Raman peaks is as follows [39]:

- The intense peak at 960 cm^-1^ was assigned to the v_1_ PO_4_ phosphate vibration.
- The peak at 1046 cm^-1^ was assigned to the v_3_ PO_4_ phosphate vibration.
- The peak at 1071 cm^-1^ was assigned to the v_1_ CO_3_ carbonate vibration (type B).
- The peak at 1244 and 1270 cm^-1^ were assigned to the parallel and perpendicular C-N vibrations relative to the collagen fibril axis, respectively
- The Raman peak at 1450 cm^-1^ was assigned to the δ (CH_2_) side chains of the collagen molecules.
- The Raman peak at 1669 cm^-1^ was assigned to the Amide I (C = O) vibration.

### 2.2 Raman Imaging Acquisitions

**Fig 2:**
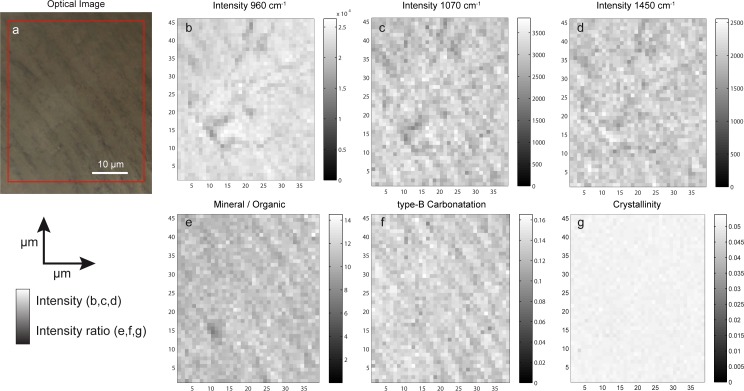
**Raman images reproduce the alternation between the bright and dark incremental lines observed under microscopy** a) optical image of the ROI showing alternating bright and dark lines of acellular cementum. b-d) spatial distribution of the intensity of Raman peaks 960cm^-1^, 1070cm^-1^ and 1450 cm^-1^; e-g) spatial distribution of the physic-chemicals parameters Mineral/Organic, type-B Carbonatation and Crystallinity

Raman images were acquired on the five perfectly ground cross-sections. From Raman images, the intensity of specific peaks and the physico-chemical parameters (PCP) were assessed and their spatial distribution reconstructed. Fig 2A represents the optical image of the ROI. Fig 2B–2G represents the spatial distribution of the Raman peaks at 960 cm^-1^, 1070 cm^-1^ and 1450 cm^-1^ and the PCP. The intensity of the color is based on the intensity of the Raman peak or the value of the PCP. The lamellar structure is reproduced by the intensity of the Raman peaks. The Mineral/Organic intensity ratio (Fig 2E), the type B carbonatation (Fig 2F) and Raman images of intensity (Fig 2B–2D) exhibit a pattern that perfectly matches the alternation between the bright and dark incremental lines observed under microscopy (Fig 2A). Conversely, the crystallinity do not reproduce the alternation between incremental lines (Fig 2G).

### 2.3 Raman acquisitions as a function of angular variations of the sample

**Fig 3:**
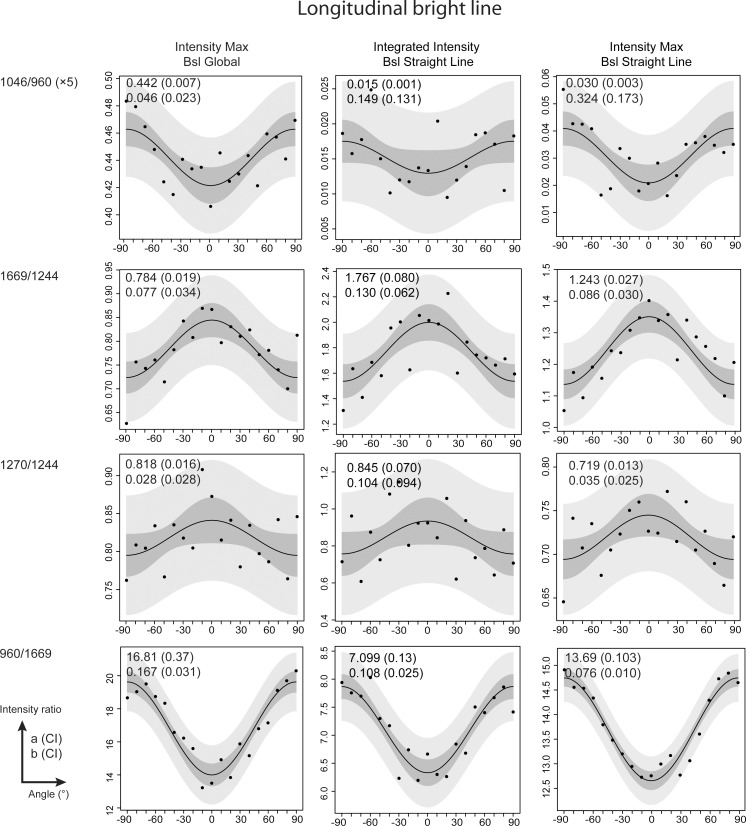
The comparison shows that the method “Int. Max + Str. Bsl” is suitable for the analysis. Comparison of the 3 methods of intensity evaluation on the fourth ratios. The comparison was done on Raman spectra taken on a bright line on a longitudinal section. Prediction and confidence interval bands are presented respectively in light and dark grey. Absolute values a and b obtained from the fitting procedure are presented in the upper corner left of each graphic. The values in brackets correspond to the confidence interval 95%.

Three extraction methods of the intensities 960, 1046, 1244, 1270 and 1669 cm^-1^ were compared in the aim to use the Eq (1) in this work. The comparison was done on the results from bright (Fig 3) and dark (**S1 Fig**) lines from a longitudinal section. The ratio 960/1669 showed similar results between the 3 methods for the dark and bright lines. Especially, the method “Integrated Int. + Str. Bsl” was in agreement with the previous works [37,38].

The ratios 1046/960, 1669/1244 and 1270/1244 showed poor sinusoidal shapes with “Integrated Int. + Str. Bsl” method for bright and dark line. For some fitted parameters b, the confidence limits were almost equal or higher than the fitted value. Two reasons were proposed to these results. Firstly, for the ratio 1046/960, the peak 1046 cm^-1^ was small and close to the baseline. Even with the integration of the intensity, the peak might be influenced by the noise. Secondly, for the ratios 1669/1244 and 1270/1244, the peaks 1244 and 1270 cm^-1^ were overlapped. So, a deconvolution of both peaks was needed. This method is not straightforward and depends on the experience of the user. For both reasons, the intensity integration method appears not suitable to evaluate the intensity of the peaks 1046, 1244 and 1270 cm^-1^. The integration was suitable to main peaks without overlapping, like υ_1_PO_4_ (960 cm^-1^) and Amide I (1669 cm^-1^).

The ratios 960/1669, 1046/960, 1669/1244 and 1270/1244 showed similar sinusoidal shape between the methods “Int. Max + Poly. Bsl.” and “Int. Max + Str. Bsl” for bright and dark lines. Both methods showed similar results for fitted parameter b concerning the ratios 960/1669, 1669/1244 and 1270/1244. But, the ratio 1046/960 did not show similar result for the fitted parameter b. A difference of a ×10 is observed for the b parameter between “Int. Max + Poly. Bsl.” and “Int. Max + Str. Bsl”.

Based on these results, the method using the maxima intensity with straight baseline was chosen for the data analysis of all Raman polarized spectra. This method was chosen because the evaluation of the intensity of 1244 and 1270 cm^-1^ did not depend on the deconvolution procedure. Moreover, the use of the straight baseline directly around the peak was preferred compared to the polynomial baseline on the entire spectrum. The use of the polynomial baseline could result in high fluctuation of intensity and depend on the user’s experience.

**Fig 4:**
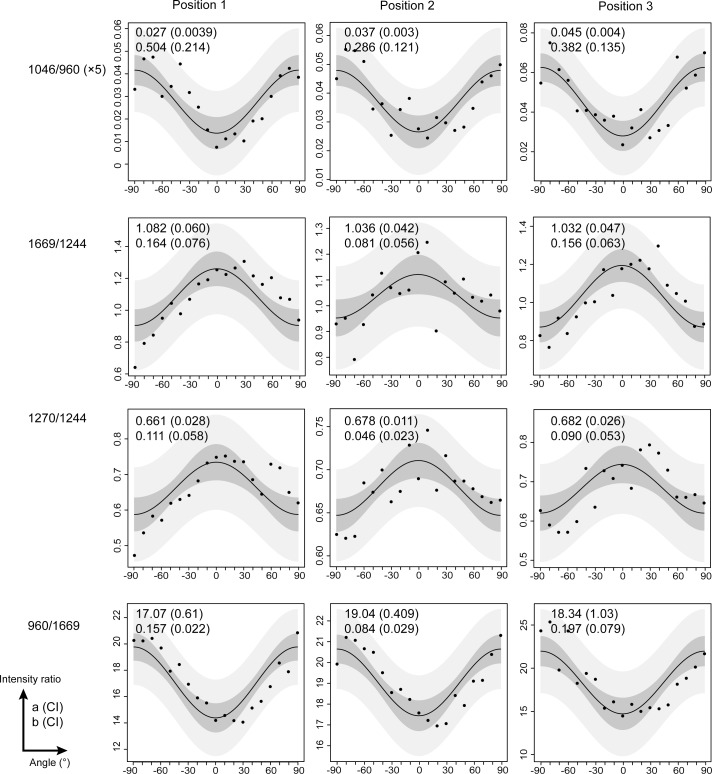
The collagen fibres and the mineral has the same orientation within the same bright line. Compares the intensity ratios 1046/960, 1669/1244, 1270/1244, and 960/1669 as a function of sample orientation of a bright line in a cross-section, in three different locations (positions 1–3). Prediction and confidence interval bands are presented respectively in light and dark grey. Absolute values a and b obtained from the fitting procedure are presented in the upper corner left of each graphic. The values in brackets correspond to the confidence interval 95%.

Intensity ratios of 1046/960 (mineral), 1669/1244, 1270/1244 and 960/1669 (collagen fibers) were studied as a function of the angle of polarization from -90° to 90°, with 10° increments at the same location. The angle of polarization defines the angle formed by the bright and dark lines and the direction of polarization. Fig 4-Position 1 shows the intensity ratios for a bright line, which were distributed symmetrically around the angle 0°. The 1046/960 and 960/1669 ratios decreased until 0°, with maximal values observed at -90° and +90°. The 1669/1244 and 1270/1244 ratios displayed the opposite behavior within the same angular range. A similar behavior was observed for dark lines (S2 Fig–Position 1). Analyses conducted on dark and bright lines resulted in the same pattern for both cementum incremental markers. The sinusoidal shape of all curves was confirmed using the fitting equation from Masic et al. [37]. The fitted absolute values (a and b) for all of the intensity ratios are shown in Fig 4. The sinusoidal shape of the ratio 960/1669 was in agreement with the results from a previous study [36].

**Fig 5:**
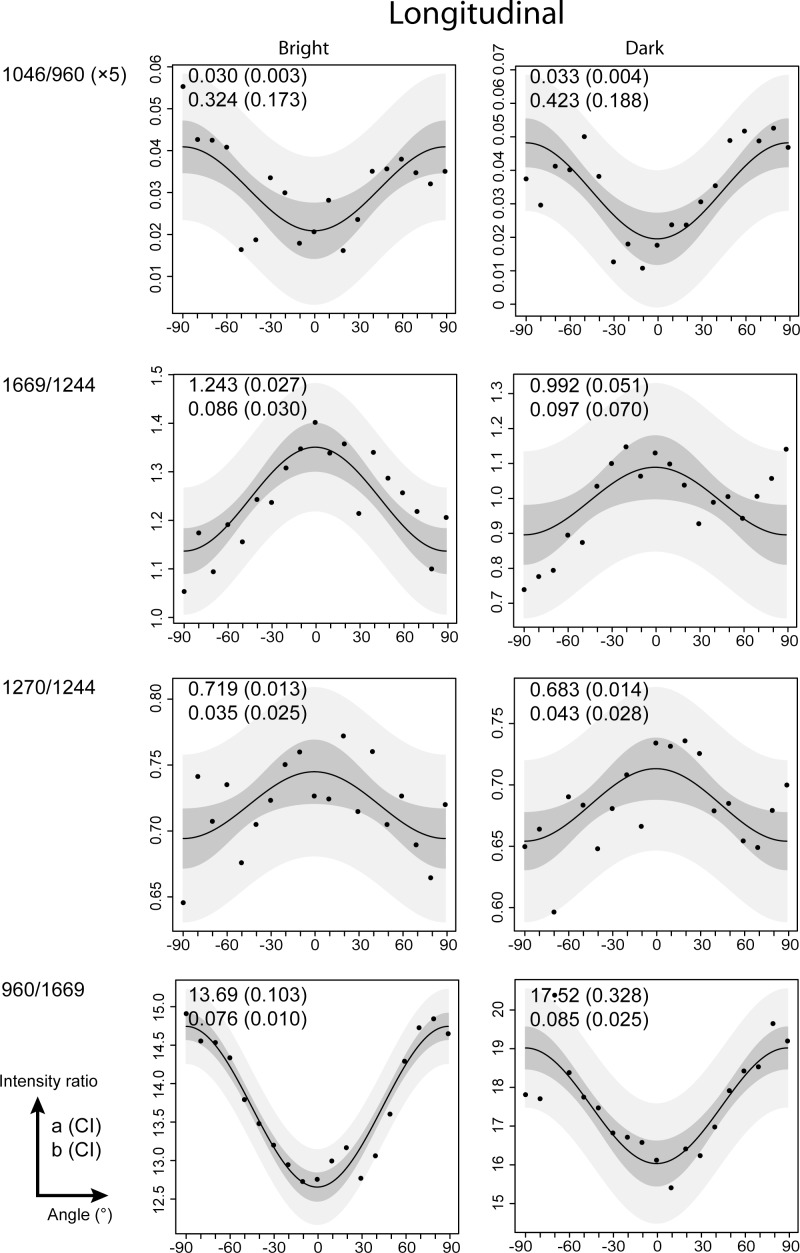
Collagen fibers and mineral crystals are parallel to the root surface in bright and dark lines of 3/5 samples. Compares intensity ratios 1046/960, 1669/1244, 1270/1244, and 960/1669 as a function of the angle of polarization of bright and dark lines observed on a longitudinal section. These results were observed in 3/5 cases. Prediction and confidence interval band are presented respectively in light and dark gray. Absolute values a and b obtained from the fitting procedure are presented in the upper corner left of each graphic. The values in brackets correspond to the confidence interval 95%.

**Fig 6:**
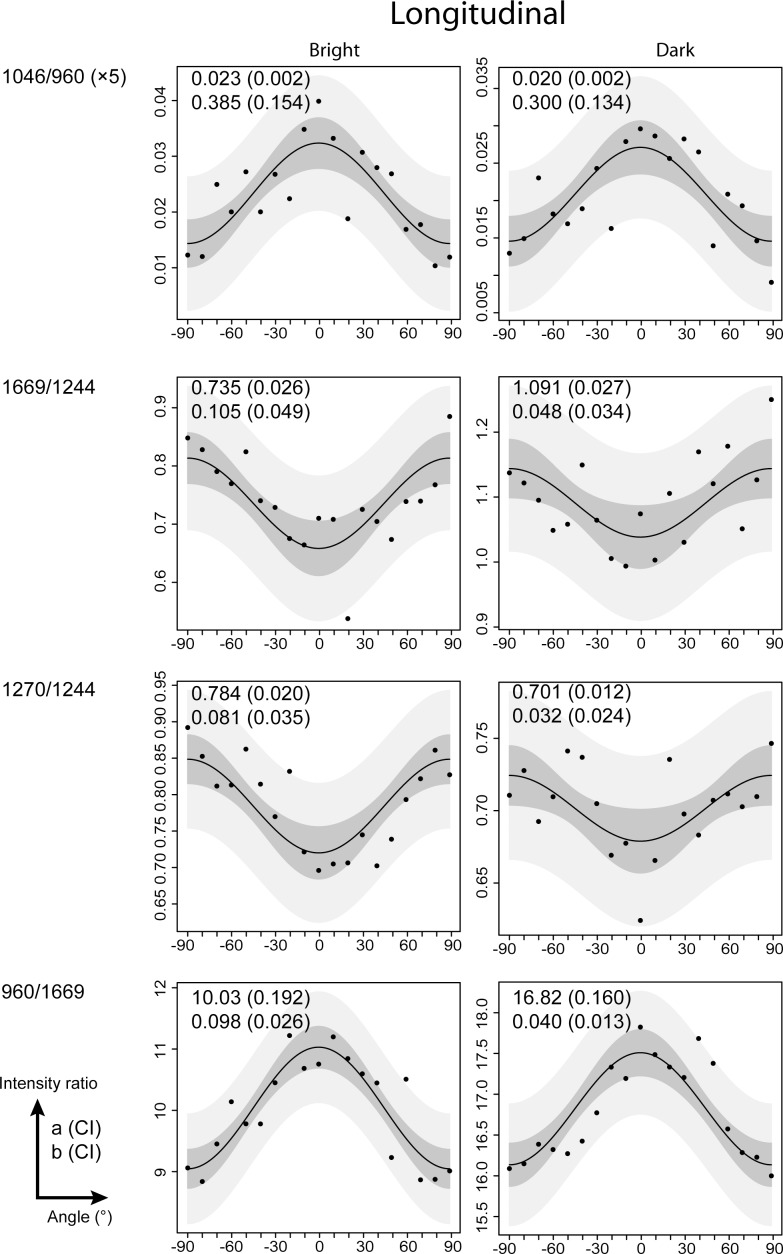
Collagen fibers and mineral crystals are perpendicular to the root surface in bright and dark lines of 2/5 samples. Compares intensity ratios 1046/960, 1669/1244, 1270/1244, and 960/1669 as a function of angle of polarization of bright and dark lines. The sample was oriented longitudinally. The sinusoidal shape has a phase shift of 90° compared to the ones in Fig 5 and S3 Fig. These results were observed in 2/5 cases. Prediction and confidence interval band are presented respectively in light and dark gray. Absolute values a and b obtained from the fitting procedure are presented in the upper corner left of each graphic. The values in brackets correspond to the confidence interval 95%.

Intensity ratios were also studied as a function of the orientation of the section (cross-section or longitudinal section). The intensity ratios and sinusoidal shapes and their fitted values were similar between the bright and dark lines in the longitudinal orientation (Fig 5). Similar results were observed for bright and dark lines in cross-section orientation (**S3 Fig**). These results were observed in 3 of the 5 teeth. Conversely, in 2 of 5 teeth, the intensity ratios followed a sinusoidal shape with a phase shift of 90° compared with the previous data. The 1046/960 and 960/1669 increased until the maximum was reached at 0°, and the minimal values were observed at -90° and +90°. The 1669/1244 and 1270/1244 ratios displayed the opposite behavior within the same angular range (Fig 6 and S4 Fig).

Moreover, Raman acquisitions were done on the same bright or dark lines at 3 different locations. The distance between each location was 50 μm. A sinusoidal profile was obtained for the 3 intensity ratios acquired at 3 different locations. These sinusoidal profiles of 1046/960, 1669/1244, 1270/1244, and 960/1669 and their fitted values (a and b) are shown in Fig 4-Positions 2 and 3 for a bright line. The results for the dark line are presented in the S2 Fig 2 Positions 2 and 3.

To summarize the results representative of all samples, the sinusoidal shapes of the intensity peak ratios (1046/960, 1669/1244, 1270/1244 and 960/1669) were similar for bright and dark lines. The sinusoidal shapes were similar within the same bright (or dark) line spaced by 50 μm. The sinusoidal shapes of bright (or dark) lines were not affected by the sample orientation (longitudinal or cross-sectional).

## III. Discussion

### 3.1 RS Chemical composition of acellular cementum

The chemical composition analysis results showed similar Raman spectra among AEFC, coronal cellular cementum and bone. According to the Raman spectra, the acellular cementum is composed of mineralized collagen fibrils. The collagen is mainly type I, and the mineral is a carbonated hydroxyapatite [14,35,40].

The main question investigated here was whether the crystallite and collagen fibril orientation and/or mineralization differed between two incremental lines. Cool et al. [23] suggested that mineral phase had the greatest influence on acellular cementum birefringence in AEFC. In our work, two Raman images were collected per sample to assess the spatial distribution of mineral and organic matter between incremental lines. Three images of Raman peak intensity (960, 1070 and 1450 cm^-1^) did reproduce the alternating bright and dark lines observed in the optical image.

The spatial distribution of the physico-chemical parameters was also assessed from the Raman images. The mineral/organic and type-B carbonatation ratio reproduced the alternating bright and dark lines, as observed by polarized microscopy. We can see that dark lines showed the highest mineral/organic ration, suggesting that they correspond to more mineralized incremental layers. These features were perfectly observed on two of our five samples, so we bear in mind that the variations in composition may be beyond the detection threshold.

### 3.2 3D Orientation of mineralized collagen fibrils in acellular cementum

Lamellar structures, with alternating bright and dark lines, are found in mammal lamellar bone, human cellular and acellular cementum, fish scuta and scales [13]. Most studies of lamellar structure have reported a predominance of a repetitive plywood-like model in which collagen fibril bundles have different orientations.

In lamellar bone, the fibrillar organization of the osteonal lamella has been the subject of numerous studies. A specific model was first described by Gebhardt in 1905 [41], in which collagen fibrils of one lamella twisted around the Haversian canal. Giraud-Guille [15] described a continuous change in orientation, with a repeated cycle of rotation of 180°, to form the twisted plywood structure in osteonal lamellae. Reznikov et al. [42,43] noted that the lamellar bone is a two-material structure in which an ordered material (aligned cylindrical fibrils rods) can be found in a disordered matrix (randomly oriented fibrils), which are both present in a lamella. Wagermaier et al. [44] found the mineral spiral around the central axis with varying degrees of tilt by applying scanning x-ray diffraction with a micron-sized synchrotron beam. Falgayrac et al. [35] showed simultaneous tilting in intra-lamellar collagen-fibril and mineral crystal orientations by RS, and this tilting vanished by moving away from the center Haversian canal. Schrof et al. [38] highlighted two different collagen fibril arrangement patterns by RS, i.e., the twisted and oscillating plywood pattern, which may coexist in the same osteon. In a fully formed cellular cementum, the intrinsic matrix fibers are arranged in a lamellar pattern, similar to the lamellar bone twisted plywood model, probably for biomechanical reasons [13]. In terms of tissue growth, Yamamoto et al. [12] observed that cementoblasts and type II osteoblast processes are quite similar and probably responsible for the twisted plywood model. Indeed, cementoblasts control the fibril arrangement via synchronized periodic changes in the orientation of their finger-like cytoplasmic processes [11–13]. Collagen fibers were observed in radial and circumferential directions relative to the longitudinal axis of the root in human cellular cementum using histology and AFM [14].

The AEFC exhibits a relatively similar pattern to bone under light microscopy (alternating bright and dark lines). However, the comparison ends here. The periodontal ligament is made of collagen fibrils, which formed thick bundles near the AEFC surface. These bundles are parallel between them and perpendicular to the cementum surface. They cross the mineralization front and enter the AEFC, where they can be followed, occasionally even into the cementodentinal junction. Raspanti et al. [6] proposed that two types of fibers can be distinguished. The main portion is represented by the thick orthogonal Sharpey’s fibers (in relation to the AEFC surface), and the other fibers, which are present in other orientations (i.e., parallel to the surface) and are sometimes called “intrinsic fibers”, are due to the branching and bending of the Sharpey’s fibers. Thus, all of these fibers are interconnected, and this network exhibits a strong biomechanical potential. Our results support this assumption.

#### Mineral crystals and collagen fiber orientation

The intensity ratios showed sinusoidal shape distributed symmetrically around the angle 0°. When maximal values of the mineral ratio (1046/960) were observed at -90 and +90°, the minimum values of the collagen fiber ratios (1669/1244; 1270/1244, 960/1669) were observed. These sinusoidal shapes, which were observed on both bright and dark lines, revealed that the mineral crystals are parallel to the collagen fibers. These results are similar to those from previous studies of bone tissue [35,37]. These sinusoidal shapes were observed across all samples in longitudinal or cross-section orientation, even among 3 points of the same incremental line. Therefore, mineral crystals and collagen fibers are always oriented in the same direction, regardless of the bright/dark line or the longitudinal/cross-section orientation.

Variability in the measures around the fitting curve can be observed in the ratio 1270/1244 of the Fig 5 for example. First, the variability may be related to the low amplitude of 1270/1244 variation (b~0.035–0.043), which was measured as a function of the orientation compared to the intensity ratio 960/1669 (b~0.076–0.085). Second, each measure plotted (Figs 3–6 and **S1**–S4 Figs) represented one spectrum. Compared with the studies of Gamsjaeger et al. [36] and Shrof et al. [38], the graphics of 960/Amide I or Amide I intensity as a function of the orientation showed a low variability of the measures around the fitted curve. In their studies, each measure was the result of averaged Raman spectra. In our study, however, each measure represented one spectrum. Nonetheless, our results from the fitting procedure were in agreement with previous studies, despite the variability in our measures.

Concerning the mineralized collagen fibers orientation, we found that collagen fibers were perpendicular to the direction of polarization and were thus mainly parallel to the root surface in 3/5 samples (Fig 5 and S3 Fig). However, in 2/5 samples, collagen fibers were parallel to the direction of polarization, which can be interpreted as being mainly perpendicular to the root surface (Fig 6 and S4 Fig). It’s important to notice that all the molecules parallel to the laser beam will produce an absolute isotropic response, we can call them parallel circumferential fibers. The anisotropy pattern observed in this study is due to the molecules tilted from the laser beam direction (tilted circumferential fibers), with highest anisotropy when they are tilted perpendicularly. Among them, those perpendiculars to the tooth surface are called Sharpey’s fibers.

In our study, two samples pointed out collagen fibers mainly oriented perpendicular to the root surface, which can be interpreted as extrinsic Sharpey’s fibers. The 3 other samples pointed out collagen fibres mainly oriented parallel to the root surface. These fibers can be interpreted as branching and bending of the extrinsic fibers, or/and randomly oriented molecules parallel to the tangential planes (in both longitudinal and cross-sections).

#### The question of the alternating lamellar pattern

Raman polarized analysis results showed that mineral crystals and collagen fibers are always oriented in the same direction both in dark and bright lines. However, if we observe the fitting curves shape (Fig 4 vs. S2 Fig), it seems that the variability around them is more noticeable in the bright lines, and so mineralized collagen fibers may be less aligned in the bright lines than in the dark lines. Parameter b mean is higher in dark lines (0,188 a.u.) versus bright ones (0,146 a.u.), reflecting a higher anisotropy degree [38].

Zoologists and zooarchaeologists called the dark lines annuli, winter lines or occasionally even rest lines, as they were thought to correspond to slowed growth periods [e.g. 14]. Based on our results, we can thus hypothesize that dark lines correspond to smaller, more mineralized and better organized lines produced during a slower (maybe in winter) period of AEFC growth.

#### Anisotropy degree

Galvis et al. [45] demonstrated that the collagen-like peptide molecule orientation determined the degree of anisotropy of amide I RS response. The Amide I band intensity (C = O stretching) showed an anisotropic response as a function of the orientation of the laser polarization. Maximal intensity was obtained when the polarization of the laser was perpendicular to the molecule and minimal when parallel. The parameter b quantifies the degree of anisotropy of amide I RS response. Schrof et al. [38] found variations in parameter b from close to isotropic to highly anisotropic for the amide I response in osteonal tissue sections, with a maximum value of parameter b of 0,17 ± 0,01 a.u. We found similar values, with a mean of 0.121 ± 0.057 a.u. and a maximum value of 0.206 a.u. If we compare these values with those obtained using the rat-tail tendon model [38,45], parameter b is ≈40 to 50% lower in human AEFC. This loss of anisotropy may be due to the presence of disordered collagen fibrils and the limited spatial resolution of the PRS, as stated by Schrof et al. [38].

### IV. Summary and Conclusion

In this work, we used the Raman spectroscopy to unveil the composition and structure of the Acellular Extrinsic Fiber Cementum. We carried out Raman imaging analysis and Polarized RS to assess the composition and orientation of mineralized collagen fibrils. We highlighted five main points:

1. The AEFC is mainly composed of carbonated hydroxyapatite and collagen type I, with a Raman spectrum response similar to that of bone and dentin.
2. The spatial distribution of peak and ratio intensities reproduced the alternating bright and dark incremental lines observed in polarized microscopy. The alternating pattern of AEFC could thus be partly related to the mineral/organic composition, with higher mineral/organic ratio in dark lines compared to bright lines. We also observed that collagen fibers seemed to be better aligned in dark lines. Our assumption, following the work of zoologists on mammals, is that the dark lines correspond to smaller, more mineralized and better organized incremental layers produced during a winter rest period.
3. Mineral crystals were always parallel to collagen fibrils.
4. Based on the results of Raman polarized acquisitions, we proposed an organization of the acellular extrinsic fiber cementum, consisting of orthogonal or radial fibers in the continuity of periodontal ligament Sharpey’s fibers and “non-orthogonal” fibers (occasionally mistakenly called intrinsic fibers), which in turn may consist of branching and bending radial fibers if we follow the Raspanti et al. model [6].
5. The parameter b indicates a mainly anisotropic material, with “orthogonal” radial mineralized Sharpey’s fibers and some “non-orthogonal” or “disordered” material consisting of the collagen fibers’ directional change through the acellular cementum.

## V. Materials and Methods

### 5.1 Preparation of samples

The five mandibular canines used in this study were extracted from five distinct patients of the Dental Service of Lille University’s Hospital. The patients gave informed written consent to participate and the study was approved by an institutional review board (DC-2008-642, French Ministry of Education and Research, DGRI/05). The initial treatment included the extraction of one or several teeth for conditions excluding periodontitis.

Teeth were cleaned and dried with acetone, and the root was embedded in an epoxy resin and dried in a vacuum chamber. The crown and upper third of the root were removed. Two sequential, 100-μm, non-decalcified cross-sections from the cervical third of the root were prepared according the ISO-9001 certified protocol for cementochronology [32], and two additional sequential longitudinal 100-μm sections were prepared. Cross-sections were made perpendicular to the exterior of the root to improve the distinction between incremental lines according to Maat et al. [46].

**Fig 7:**
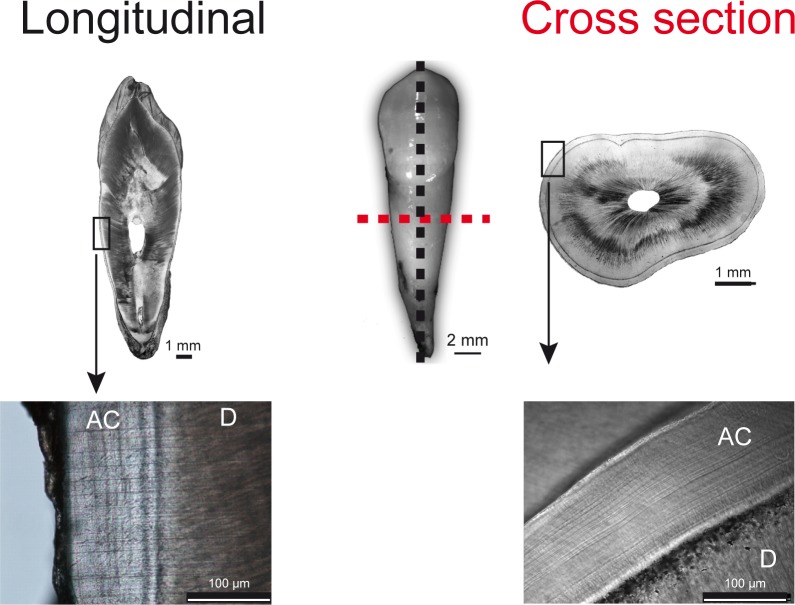
Longitudinal and cross sections (100μm) of acellular extrinsic fibers cementum. AC: Acellular Cementum, D: Dentin

One longitudinal section and one cross-section were mounted on slides with Canada basalm. Preliminary observations were made using a Nikon Eclipse 50i microscope at 200X or 400X magnification. Segments that showed readable cementum lines were captured as JPEG images with a Nikon DS Fi1 fitted on the microscope (Fig 7). Readings of selected segments were completed using Adobe Photoshop CS5. Then, the same sections were analyzed by Raman spectroscopy.

### 5.2 Raman Spectroscopy

Raman spectra were acquired using a LabRAM Raman microspectrometer (Jobin-Yvon, France) [35]. The instrument is equipped with a xyz motorized stage and a diode laser at 632.8 nm. x, y and z represent the axes of an orthonormed system, where the z-axis is perpendicular to the sample surface and parallel to the laser beam. The spectrometer uses a high-precision Piezo translator and feedback signal to automatically track and adjust the laser focus on the sample, which ensures a perfect focus for each measurement. A CCD detector (1024×256) was used to collect the Raman signal. The acquisitions were done with an objective of ×100 (numerical aperture 0.9). Raman spectra were acquired in the 900–1700 cm^-1^ spectral range.

### 5.3 Raman Imaging Analysis

Raman imaging was carried out using the point-by-point imaging mode (Pt-Img). The laser beam was focused perpendicular to the sample surface. Acquisition time was set to 30sec for each spectra. The laser beam was stepped in two dimensions (x and y), and a spectrum was recorded at each position (x,y). The Pt-Img mode generated x×y spectra.

Prior to Raman imaging, regions of interest (ROI) of acellular cementum were selected on both the cross-section and the longitudinal section under light microscopy. The same ROIs were located on the perfectly polished cross-section with the Raman microscope. Bright and dark lines were analyzed using the Pt-Img mode at a pixel size of 1 μm. Pt-Img analyses were done to evaluate compositional variations across the ROI, which provided a spectral mapping analysis. Two Raman images per sample were analyzed. The size of the Raman image varied between 24×24 to 39×46 pixels.

The intensity of a Raman peak is sensitive to the compositional variation and orientation of the active group within the sample [47]. A scrambler was used to eliminate variations in intensity related to the orientation of molecules during the Pt-Img analysis. Therefore, the intensity variations were related only to the compositional variations within the active group. Variations in composition and structure were evaluated with three physico-chemical parameters (PCP) [48], including the mineral/organic ratio, which corresponds to the intensity ratio between the ν_1_ PO_4_^3-^ (960 cm^-1^) to the δ (CH_2_) side chains of collagen molecules (1450 cm^-1^) peaks. The mineral/organic ratio reflects the amount of mineral per amount of organic matrix. The type-B carbonate substitution corresponds to the intensity ratio between B-type CO_3_^2-^ (1070 cm^-1^) and the ν_1_ PO_4_^3-^ peak (960 cm^-1^). The type-B carbonate substitution reflects the amount of B-type CO_3_^2-^ per amount of mineral. The crystallinity corresponds to the inverse of the full width at half maximum of the ν_1_ PO_4_^3-^ (960 cm^-1^) and reflects the mineral crystal size and perfection. This parameter is increased during mineralization from initial amorphous calcium phosphate phases to well-ordered apatite forms.

### 5.4 Raman polarized acquisitions as a function of the angle of polarization

**Fig 8:**
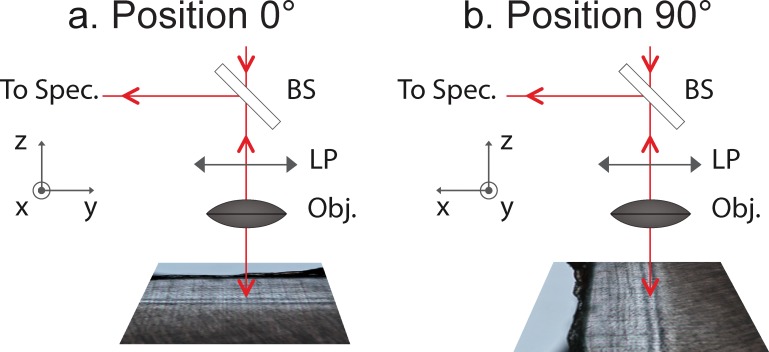
Representation of the Raman experimental setup carried out on the AEFC samples as a function of sample orientation. The angle of polarization defines the angle formed by the bright and dark lines and the direction of polarization. In the position (a), the bright and dark lines are parallel to the direction of polarization which corresponds to the angle of polarization 0°; in the position (b) the bright and dark lines are perpendicular to the direction of polarization which corresponds to the angle of polarization +90° (or -90°); (BS) Beam splitter; (LP) linear polarizer; (Obj) objective; and (To Spec.) to spectrograph.

Raman polarization analyses were performed on each longitudinal and cross-section from the five samples (Fig 7). Acquisition time was set to 5 min (60sec averaged 5 times) per spectrum. Each sample was mounted on a goniometer. The direction of polarization was kept fixed while the sample was rotated at different angles. The Fig 8 describes the experimental set-up. The angle of polarization defines the angle formed by the bright and dark lines and the direction of polarization. The angle of polarization was defined as 0° when the bright and dark lines are parallel to the direction of polarization (Fig 8A). The angle was defined as -90° (or +90°) when the bright and dark lines were perpendicular to the direction of polarization (Fig 8B). The sample was rotated in steps of 10° to obtain polarizations at different angles ranging from -90° to +90°. The intensity variations of Raman peaks 960, 1046, 1244, 1270 and 1669 cm^-1^ were evaluated as a function of angular variations in the sample at the same point. The variations of the intensity ratios (1046/960, 1669/1244, 1270/1244 and 960/1669) were calculated as functions of the angle of polarization. Each measure at each angle plotted is representative of one spectrum. A previous work demonstrated that these ratios were sensitive to the apatite crystal and collagen fibril orientation [35,47]. The ratio 1046/960 is sensitive to the apatite crystal orientation. The ratios 1669/1244, 1270/1244 and 960/1669 [35,36] are sensitive to the collagen fibrils’ orientation. Bright and dark lines were analyzed for each longitudinal section and cross-section. This procedure was done at 3 different locations of the same line (bright or dark). The distance between each location was 50 μm.

### 5.5 Data analysis

#### Raman imaging

Raman spectra were processed using the Labspec software (HORIBA, Jobin-Yvon, France). A Savitzky−Golay smoothing filter (filter width: 2; and polynomial order: 2) was applied. The intensity is integrated over defined Raman shift regions in the spectrum using a sum filter [37,38]. The filter calculates the intensities within the chosen borders and the background is subtracted by taking the baseline from the first to the second border [37,38]. This procedure was applied on the Raman peaks 960 cm^-1^ (peak-ROI 900–990 cm^-1^), 1070 cm^-1^ (peak-ROI 1052–1092 cm^-1^) and 1669 cm^-1^ (peak-ROI 1610–1700 cm^-1^). The intensity, the PCPs and their spatial distributions were calculated using the PLS toolbox (v8.2, Eigenvector Research, Inc., Wenatchee, WA, USA) and a custom-developed script in Matlab R2013a (Mathworks Inc, Natick, MA, USA).

#### Raman polarized acquisition

In our group, the peak intensity maxima and a polynomial baseline correction over the entire spectrum was used to assess the intensity of 960, 1046, 1244, 1270 and 1669 cm^-1^ [35]. In previous works, the intensity of peaks was assessed by the method of intensity integration over defined Raman shift regions using a sum filter with straight baseline between peak-ROI [37,38]. This method was applied on intense peaks 960 and 1669 cm^-1^ and used in the Eq (1). To use the Eq (1) in this work, the method of intensity integration cannot be strictly applied especially for the overlapped peaks 1244 and 1270 cm^-1^. Thus, three extraction methods of the intensity 960, 1046, 1244, 1270 and 1669 cm^-1^ were compared to choose the most suitable, in the aim of using the Eq (1) in this work.

1. Intensity maxima with polynomial baseline (Int. Max + Poly. Bsl.): polynomial baseline correction (degree 4) is done on the entire spectrum and the peak maxima is extracted. A special care was taken that polynomial baseline correction do not distort the Raman bands. [35]
2. Integrated intensity with straight baseline (Integrated Int. + Str. Bsl): the sum of the whole area beneath the peaks with a straight baseline between the chosen spectral peak-ROI [37,38]. The peaks 1244 and 1270 cm^-1^ were deconvoluted with a Gaussian function with Labspec software.
3. Intensity maxima with straight baseline (Int. Max + Str. Bsl): the maxima peak intensity with straight baseline between the spectral peak-ROI. This method is combination of the both above.

This comparison helped to determine the optimal method to evaluate peak intensities and use them in the Eq (1). The comparison was based on the results from the following fitting procedure.

The intensity ratios (1046/960, 1270/1244, 1669/1244, 960/1669) showed a sinusoidal evolution as function of the angle of polarization. The ratios were fitted according to the following equation [36,37]:

R=a(1+b(cos2(x−c))) (1)

where R is the intensity ratio, a is the mean intensity of all scans, b is the amplitude of the fitting curve, x is the angle of polarization (radian) and c is the phase shifting (radian). The parameters and their standard deviations were estimated with a nonlinear least-squares fitting procedure using R [49,50]. In lamellar bone, Schrof et al. [38] provided evidence of a correlation between amide I intensity and the degree of anisotropy of the Raman response, thus highlighting the role of parameter b, which is a reliable indicator of fibril orientation.

## Supporting Information

S1 Fig The comparison shows that the method “Int. Max + Str. Bsl” is suitable for the analysis. Comparison of the 3 methods of intensity evaluation on the fourth ratios. The comparison was done on Raman spectra taken on a dark line on a longitudinal section. Prediction and confidence interval bands are presented respectively in light and dark grey. Absolute values a and b obtained from the fitting procedure are presented in the upper corner left of each graphic. The values in brackets correspond to the confidence interval 95%.
(TIF)

S2 Fig The collagen fibres and the mineral has the same orientation within the same bright line. compares the intensity ratios 1046/960, 1669/1244, 1270/1244, and 960/1669 as a function of sample orientation of a dark line in a cross-section, in three different locations (positions 1–3). Prediction and confidence interval bands are presented respectively in light and dark grey. Absolute values a and b obtained from the fitting procedure are presented in the upper corner left of each graphic. The values in brackets correspond to the confidence interval 95%.
(TIF)

S3 Fig Collagen fibers and mineral crystals are parallel to the root surface in bright and dark lines of 3/5 samples. compares intensity ratios 1046/960, 1669/1244, 1270/1244, and 960/1669 as a function of the angle of polarization of bright and dark lines observed in a cross-section. These results were observed in 3/5 cases. Prediction and confidence interval band are presented respectively in light and dark grey. Absolute values a and b obtained from the fitting procedure are presented in the upper corner left of each graphic. The values in brackets correspond to the confidence interval 95%.
(TIF)

S4 Fig Collagen fibers and mineral crystals are perpendicular to the root surface in bright and dark lines of 2/5 samples. compares intensity ratios 1046/960, 1669/1244, 1270/1244, and 960/1669 as a function of angle of polarization of bright and dark lines. The sample was oriented along the cross-section. The sinusoidal shape has a phase shift of 90° compared to the ones in Fig 5 and S3 Fig. This result is observed in 2/5 cases. Prediction and confidence interval band are presented respectively in light and dark grey. Absolute values a and b obtained from the fitting procedure are presented in the upper corner left of each graphic. The values in brackets correspond to the confidence interval 95%.
(TIF)

S1 Table **Raw data of Raman acquisitions presented in Figs 1–6 and S**1**–S**4 **Figs** Sheet 1 in S1 Table: contains Polarized Raman spectra of Fig 1; Sheet 2 in S1 Table: Raman images of the intensity peaks, intensity ratios and crystallinity as function of the pixel number. These data correspond to the Fig 2; Sheet 3 to 6 in S1 Table: intensity ratios 1046/960, 1669/1244, 1270/1244 and 960/1669 as function of the angle of rotation and the data analysis procedure. These data are presented in Fig 3 and S1 Fig; Sheet 7 to 12 in S1 Table: intensity ratios 1046/960, 1669/1244, 1270/1244 and 960/1669 as function of the angle of rotation and the position within the same bright or dark line. These data are presented in the Fig 4 and the S2 Fig; Sheet 13 to 16 in S1 Table: intensity ratios 1046/960, 1669/1244, 1270/1244 and 960/1669 as function of the angle of rotation, the orientation of the sample and bright or dark line. These results are presented in 3/5 samples and presented in the Fig 5 and S3 Fig; Sheet 17 to 20 in S1 Table: intensity ratios 1046/960, 1669/1244, 1270/1244 and 960/1669 as function of the angle of rotation, the orientation of the sample and bright or dark line. These results are observed in 2/5 samples and presented in the Fig 6 and S4 Fig.
(XLSX)
