# Correction: New Insights on the Composition and the Structure of the Acellular Extrinsic Fiber Cementum by Raman Analysis

**DOI:** 10.1371/journal.pone.0174080

**Published:** 2017-03-10

**Authors:** Thomas Colard, Guillaume Falgayrac, Benoit Bertrand, Stephan Naji, Olivier Devos, Clara Balsack, Yann Delannoy, Guillaume Penel

There is an error in affiliation 3 for author Stephan Naji. Affiliation 3 should be: New York University-CIRHUS, New York City, NY, United States of America.

The second affiliation for author Stephan Naji is not indicated. Stephan Naji is also affiliated with: Univ. Côte d’Azur, CNRS, UMR 7264-CEPAM, France.

The following information is missing from the Acknowledgments section: The authors would also like to thank the Agence National de la Recherche (project CemeNTAA ANR-14-CE31-0011) for organizational help.
