# Supplementary figures and images for: New Insights on the Composition and the Structure of the Acellular Extrinsic Fiber Cementum by Raman Analysis
Source: PLoS One. 2016 Dec 9;11(12):e0167316. doi: 10.1371/journal.pone.0167316 (PMC5147880; doi:10.1371/journal.pone.0167316)

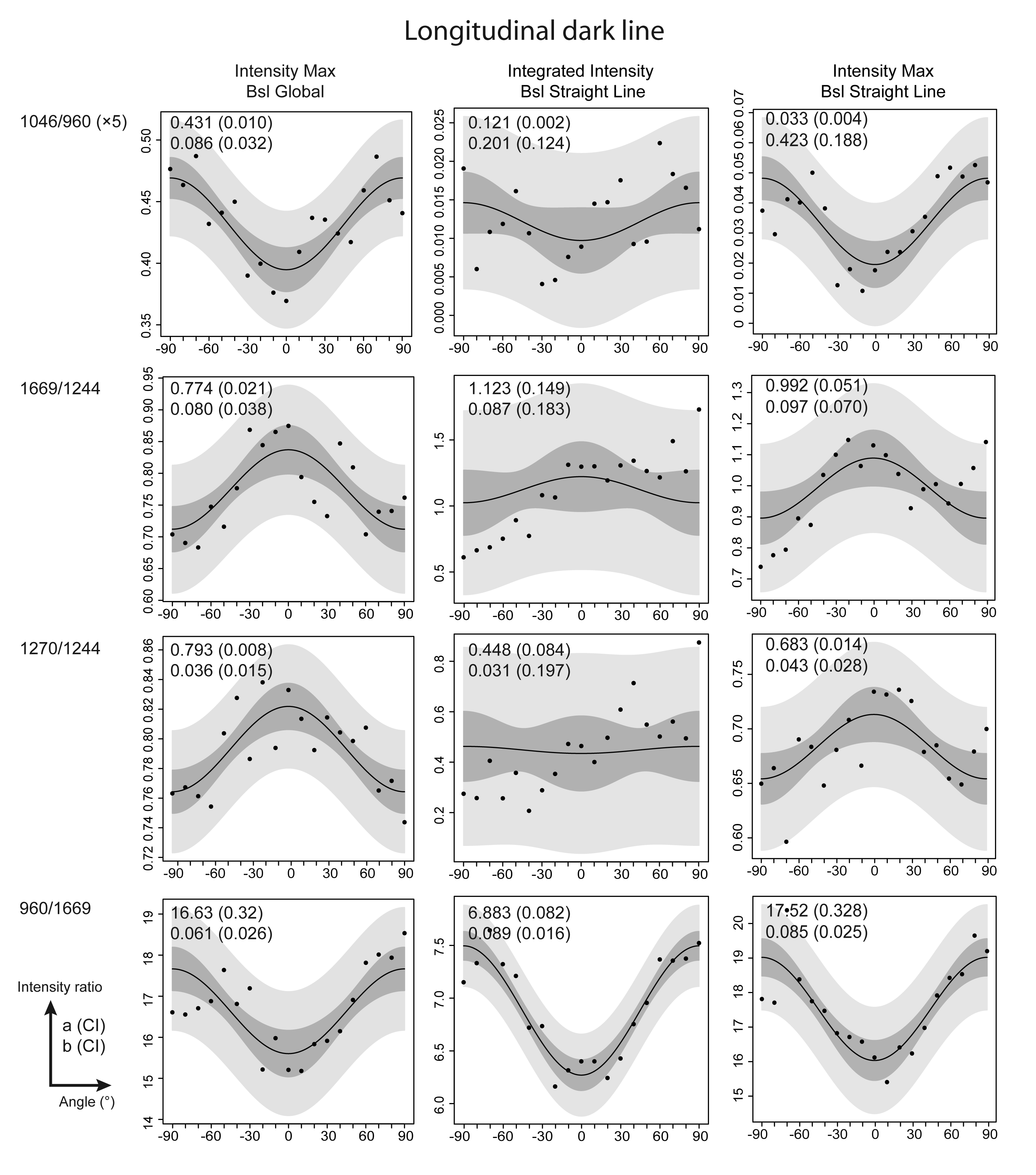

Supplement: S1 Fig — Comparison of the 3 methods of intensity evaluation on the fourth ratios. The comparison was done on Raman spectra taken on a dark line on a longitudinal section. Prediction and confidence interval bands are presented respectively in light and dark grey. Absolute values a and b obtained from the fitting procedure are presented in the upper corner left of each graphic. The values in brackets correspond to the confidence interval 95%. (TIF) [file pone.0167316.s001.tif]

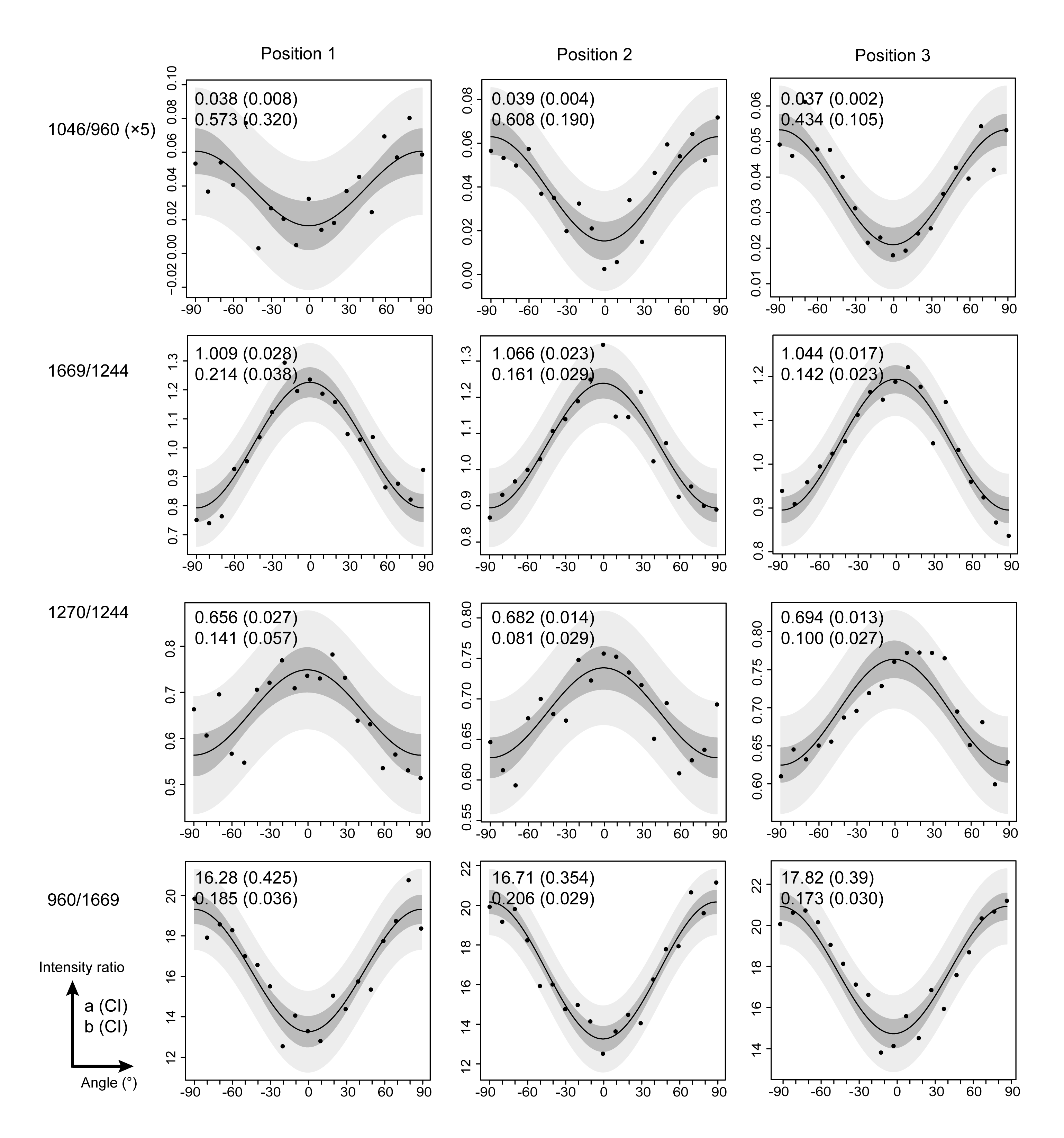

Supplement: S2 Fig — compares the intensity ratios 1046/960, 1669/1244, 1270/1244, and 960/1669 as a function of sample orientation of a dark line in a cross-section, in three different locations (positions 1–3). Prediction and confidence interval bands are presented respectively in light and dark grey. Absolute values a and b obtained from the fitting procedure are presented in the upper corner left of each graphic. The values in brackets correspond to the confidence interval 95%. (TIF) [file pone.0167316.s002.tif]

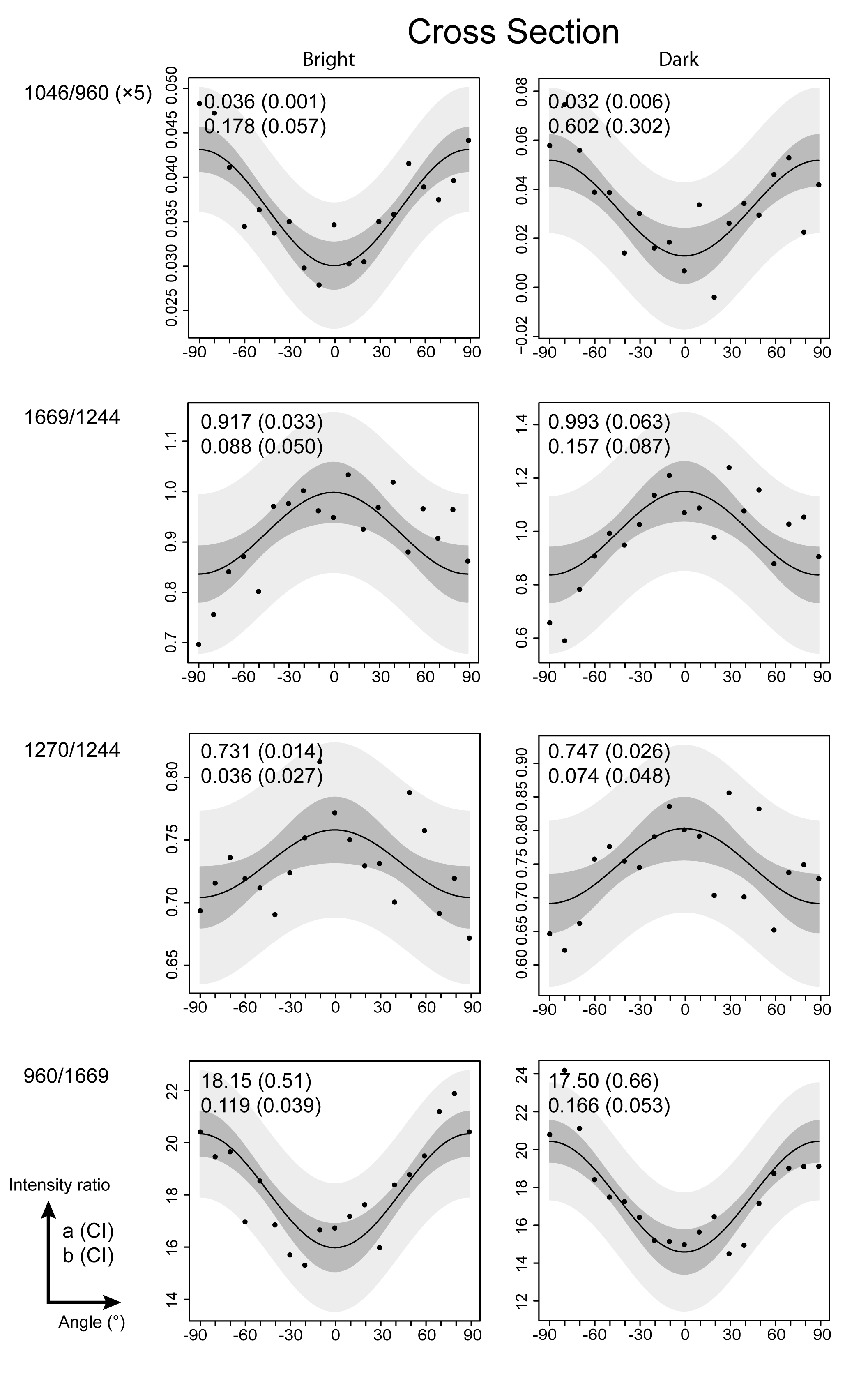

Supplement: S3 Fig — compares intensity ratios 1046/960, 1669/1244, 1270/1244, and 960/1669 as a function of the angle of polarization of bright and dark lines observed in a cross-section. These results were observed in 3/5 cases. Prediction and confidence interval band are presented respectively in light and dark grey. Absolute values a and b obtained from the fitting procedure are presented in the upper corner left of each graphic. The values in brackets correspond to the confidence interval 95%. (TIF) [file pone.0167316.s003.tif]

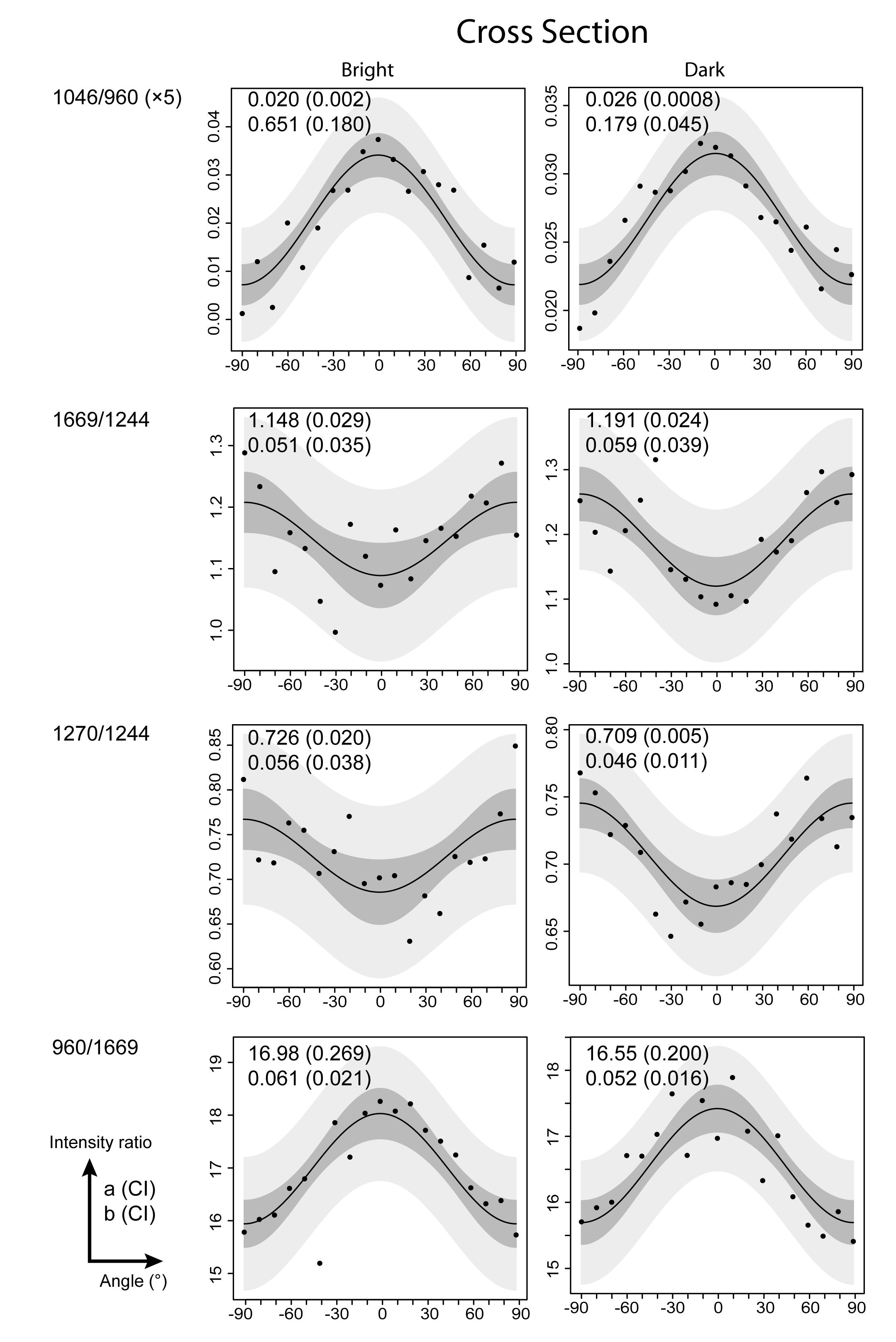

Supplement: S4 Fig — compares intensity ratios 1046/960, 1669/1244, 1270/1244, and 960/1669 as a function of angle of polarization of bright and dark lines. The sample was oriented along the cross-section. The sinusoidal shape has a phase shift of 90° compared to the ones in Fig 5 and S3 Fig. This result is observed in 2/5 cases. Prediction and confidence interval band are presented respectively in light and dark grey. Absolute values a and b obtained from the fitting procedure are presented in the upper corner left of each graphic. The values in brackets correspond to the confidence interval 95%. (TIF) [file pone.0167316.s004.tif]
